# Feline Cognition and the Role of Nutrition: An Evolutionary Perspective and Historical Review

**DOI:** 10.3390/ani14131967

**Published:** 2024-07-03

**Authors:** Allison P. McGrath, Daniel J. Horschler, Leslie Hancock

**Affiliations:** Hill’s Pet Nutrition, Topeka, KS 66603, USA; daniel_horschler@hillspet.com (D.J.H.); leslie_hancock@hillspet.com (L.H.)

**Keywords:** domestic cat, cognition, nutrition, behavior, cognitive dysfunction syndrome, aging, pet food, diet

## Abstract

**Simple Summary:**

The cognitive health of cats is closely related to their well-being and quality of life. Feline cognition encompasses cats’ ability to receive, process, and respond to sensory information. Although research surrounding feline cognition has been increasing in recent years and has led to novel discoveries about cats’ cognitive abilities, there is still much to be learned about this topic. This review discusses the evolutionary history of the domestic cat and how it became a beloved companion animal, describes what is known about cats’ cognitive function based on groundbreaking research and cognitive evaluations, and investigates the impact of nutrition on cognitive health, particularly age-related cognitive decline. By considering what is currently known about the mental health of cats and how their cognition is affected by external factors, and by identifying and narrowing gaps in our knowledge, we can help improve the welfare of and quality of life of cats.

**Abstract:**

Research into cognition in cats and the impact of nutrition on cat cognitive health lags behind that in dogs but is receiving increased attention. In this review, we discuss the evolutionary history of the domesticated cat, describe possible drivers of domestication, and explore the interrelationships between nutrition and cat cognition. While most cat species are solitary, domesticated cats can live in social groups, engage in complex social encounters, and form strong attachments to humans. Researchers have recently started to study cat cognition using similar methods as those developed for dogs, with an initial primary focus on perception and social cognition. Similar to dogs, cats also show cognitive and behavioral changes associated with stress and aging, but these signs are often gradual and often considered a consequence of natural aging. Despite the fundamental role of nutrition in cognitive development, function, and maintenance, research into the association between nutrition and cognition in cats is only preliminary. Ultimately, additional research is needed to gain a full understanding of cat cognition and to explore the role of nutrition in the cognitive health of cats to help improve their welfare.

## 1. Introduction

The domesticated cat is one of the world’s most popular companion animals. Despite their popularity, relatively little is known about feline cognition, the evolution of cat behavior, and the role that domestication has played in shaping the mental health and capabilities of cats, especially compared to what is known about the cognitive abilities of our other popular companion animal, the dog [1,2,3]. Similarly, little is known about the social behavior of cats, particularly in the context of human interaction and the home environment, and the lack of research in this area is possibly due to a widely held misconception that domestic cats are not a social species [4]. While research into cat cognition has lagged behind that of dogs, which has exploded in recent decades [5], work investigating the perceptual abilities and sociality of cats, including their attachment to humans, has grown in recent years. Still, many cognitive skills, social tendencies, and behavioral patterns in cats remain largely unexplored.

Although the totality of evidence suggests that domestic cats have developed a range of behaviors and mechanisms that facilitate their interactions with humans, cats have frequently been portrayed in a negative light in popular culture and the press, and they are often depicted as selfish, unfeeling, or manipulative, revealing deep-rooted societal biases that may have contributed to limited research on the topic [3,6,7]. Cat research may also be impeded by societal bias stemming from negative associations in folklore and mythology, including the association of cats with witchcraft, misogyny, and their occasionally ambivalent relationship with human society [8]. Ultimately, these associations may affect not only the incentive for research into cat cognition but also the care and welfare of pet, stray, or feral cats [3,9,10]. Therefore, a greater understanding of cat cognition could have important implications for improving cat welfare in both the home and in shelters and enhancing human–cat interactions.

The provision of comprehensive health care for cats necessitates an understanding of cats’ physical, emotional, and cognitive health [11]. Given the potential for nutrition to impact each of these components (see Section 5), a greater understanding of the influences of feline nutrition on cognitive function may also aid in our ability to positively influence feline health and quality of life.

The objective of this review is to consider how the study of feline cognition has evolved, its status today, and the influences of nutrition on cognition in cats. For the purposes of this review, cat cognition is defined as the ways cats can receive sensory information, process it, retain it, and use it to guide behavior [3,12].

## 2. Evolutionary History of Cats

### 2.1. Overview of the Feline Lineage

It is estimated that placental mammals diverged from an ancestor that lived about 105 million years ago [13]. The order Carnivora diverged from its closest relative, the Pholidota (pangolins), about 78 million years ago and cat-like carnivores, including cats, hyenas, mongoose, and civets, split from dog-like carnivores 40 to 55 million years ago [13,14,15,16]. Saber-toothed cats appeared about 35 million years ago, but modern felids arose about 10.2 million years ago [13].

Felidae, which includes 37 modern species, has been considered the world’s most successful, widespread, and adaptive carnivore family, with native species existing on all continents other than Australia and the poles [13]. The Felis lineage diverged from other feline lineages 6.2 million years ago and includes four species of small cats that originated near the Mediterranean basin, including the jungle cat (*Felis chaus*), the black-footed cat (*F. nigripes*), the sand cat (*F. margarita*), and the wildcat (*F. silvestris*) [8,17].

Evidence suggests that domestic cats descended from *Felis silvestris*, a polytypic wild species composed of three or more distinct interfertile subspecies, including *Felis s. silvestris* in Europe, *F. s. lybica* in Africa and Western Asia, and *F. s. ornata* in the Middle East and central Asia, and, possibly, from the Chinese desert cat *F. s. bieti* [8,17,18]. In fact, the domestic cat (*Felis catus*) is occasionally considered a subspecies of *F. silvestrus* [18]. However, the International Union for Conservation of Nature Cat Classification Task Force considers the domestic cat to be its own distinct species [19].

Difficulty in distinguishing morphological differences between feral domestic cats and local wildcats, in addition to occasional interbreeding, has generated some disagreement about which subspecies gave rise to the domestic cat [8]. Surviving subpopulations of *F. s. libyca* wildcats in remote desert areas in Israel, United Arab Emirates, and Saudi Arabia were found to be almost genetically indistinguishable from domestic cats, suggesting that the original progenitors of the domestic cat came from *F.S. libyca* residing in this geographic region [8].

### 2.2. Social and Behavioral Characteristics

Most members of the Felidae family lead solitary lives and only engage in social behavior when mating or raising young [3,20]. Still, both cheetahs and African lions live in social groups, with African lions exhibiting relatively more complex forms of sociality, at least regarding their fission–fusion group structure and egalitarian female prides [21]. Cross-species studies evaluating personality structures in different species of felids (domestic cats, Scottish wildcats, clouded leopards, snow leopards, and African lions) have reported striking similarities among species, suggesting that personality structures in felids may have evolved early in Felidae [22].

Unlike other *F. silvestris* species, social groups of *Felis catus* often form when there is a sufficient concentration of prey or other food [21,23]. Because all other subspecies of *F. silvestris* are exclusively solitary, even in the presence of sufficient concentrations of food, group-living in *Felis catus* likely occurred during the domestication process.

### 2.3. Nutritional Considerations

Members of the Felidae family are obligate carnivores and are limited in their ability to exploit other sources of food due to a variety of nutritional adaptations [21,24]. In fact, the Felidae family is the only Carnivora family in which all members can be considered truly carnivorous [25].

In general, the diet of wild cats consists of animal tissue, with birds and small mammals being the most common prey, depending on the ecosystem [26]. For example, surveys of the contents of feral cats’ stomachs indicate that small mammals, including mice, rats, and rabbits, comprise the bulk of cats’ diets, while birds, frogs, reptiles, and insects are eaten less frequently [25]. Given the small size of the prey, feral cats generally eat several small meals each day to meet their nutritional requirements.

Cats have adapted to a long history of consuming prey as their sole food source. As a result, the protein requirement of cats is substantially greater than that of other species, with kittens reported to require 1.5 times more protein than that of the young of omnivorous species and adults requiring 2 to 3 times more protein than adults of omnivorous species, including chickens and pigs [24,27]. Cats have a higher need for protein as an energy source and require greater levels of specific amino acids and related compounds in their diet, including taurine, arginine, methionine, and cysteine, than omnivores [27,28]. These amino acid compounds are not stored in large amounts in the body, and cats cannot synthesize them in sufficient quantities. Deficiencies in these amino acids can result in multiple clinical signs and adverse outcomes. For example, prolonged taurine deficiency can result in blindness, reproductive failure or neonatal loss, and the development of dilated cardiomyopathy [27,28,29]. Arginine deficiency can result in hyperammonemia, leading to salivation, neurologic abnormalities, hyperesthesia, emesis, tetany, and coma, in severe cases [27,30,31].

In terms of taste preferences, researchers have found that cats are insensitive to salts and may not respond to sugars in a behaviorally meaningful way, as evidenced by their inability to distinguish between water and water with dissolved sucrose [26,32,33]. It is possible that this adaptation may permit a more sensitive perception of taste in meat, such as monophosphate nucleotides, which may indicate time since the death of the prey [26].

Cats also have greater dietary requirements for certain vitamins than many other mammals due to the reduced activity of enzymes involved in synthesis pathways of vitamins A and D and niacin [24]. Cats are unable to convert sufficient beta-carotene to retinol, the active form of vitamin A [34]. Therefore, they must obtain vitamin A in its biologically active form from animal tissues in their diet. Although vitamin A plays a vital role in the maintenance of vision, the growth of bone and muscle, and reproduction, vitamin A deficiency is rare in cats and typically only develops in cats with severe liver failure or gastrointestinal diseases that result in the malabsorption of fat.

Similarly, cats have a reduced ability to synthesize vitamin D via dermal photosynthesis in comparison with other mammalian species because they are deficient in 7-dehydrocholesterol, a precursor required for its synthesis [27,35]. However, because vitamin D is found at high concentrations in the liver and fatty tissues of their prey, cats typically meet their vitamin D needs through their diet [24,27]. While vitamin D plays a critical role in phosphorus and calcium homeostasis and the absorption, retention, and deposition of calcium in the bone, vitamin D deficiency is rare and develops slowly. The supplementation of both vitamin A and vitamin D should be approached carefully given the toxic effects of high concentrations of both vitamins.

In most animals, niacin content is determined by the sum of dietary nicotinamides and the endogenous nicotinic acid synthesized from tryptophan [24]. Although all the enzymes involved in niacin synthesis are present in cats, the activity of picolinic carboxylase, an enzyme that catalyzes the degradation of the precursor of nicotinic acid into acetyl CoA and CO_2_, is very high, limiting the availability of this precursor and the ability to synthesize nicotinic acid. However, meat has high concentrations of dietary nicotinamides, and because cats consume a diet of animal tissue, they typically do not need to produce much niacin from tryptophan.

Cats show several physiological adaptations that demonstrate their long history of low carbohydrate and high protein intake [27]. Cats lack salivary amylase, the enzyme that initiates carbohydrate digestion [27,36]. They also have reduced activity of intestinal disaccharidases that break down carbohydrates in small intestines [27]. They also have a limited ability to minimize hyperglycemia after significant glucose intake and are more likely to store additional starch in the diet as fat rather than glycogen due to reductions in hepatic glycogen synthesis. In contrast, cats have high activities of enzymes involved in protein catabolism and gluconeogenesis [37,38].

The adaptations discussed above are a representation of metabolic efficiency. Over thousands of years, cats’ prey-based diets resulted in selective pressure, favoring cats with the most efficient ability to process high levels of protein, while pressure to efficiently process carbohydrates and synthesize nutrients like arginine, taurine, niacin, vitamin A, and vitamin D was lacking. As a result, today, domestic cats are efficient metabolizers of amino acids but have a more limited ability to metabolize carbohydrates and synthesize arginine, taurine, and certain vitamins. However, even with these evolutionary adaptations, studies have shown that domestic cats are able to effectively digest and absorb starch included in the diet [38]. Domestic cats can still utilize carbohydrates to meet their glucose and energy needs and they can adapt to diets with extensive variations in macronutrient content [39].

## 3. Cat Domestication

### 3.1. History of Domestication

The evolutionary split between wild and domestic cats is thought to have occurred approximately 10,000 years ago, when manmade stores of grain in the Near East induced the expansion of the house mouse from its origins in India [18,21,40]. The species *F. s. lybica* was presumably attracted by high concentrations of mice and other rodents and began to specialize in hunting around and within human settlements, ultimately becoming reproductively isolated from its wild counterparts [21]. Although it is likely that rodent hunting in human settlements occurred at night, natural selection in ancestral cats may have favored the bolder individuals who were more tolerant of humans, allowing them to exploit both the prey and shelter available in villages [8,41].

While this initial stage of cat domestication was likely largely self-directed, these reproductively isolated populations may have enabled the pet-keeping that drove the later stages of domestication [8]. It was these cats who established the foundation for the permanent, more urban domesticated cat populations that increasingly relied on humans for food and shelter [8]. The proximity of cats to human settlements was also mutually beneficial, with cats protecting the food supply of humans and humans providing a food source and shelter for cats [42].

The history of domestication in cats appears to differ in some ways from canine domestication, which occurred thousands of years earlier and was likely driven, at least in the later stages of the domestication process, by genetic selection to address human needs, such as hunting. In contrast, cats appear to have undergone few genetic modifications during their process of domestication, possibly because farmers found the innate behaviors of cats highly desirable [42]. Therefore, many of the naturally occurring behaviors of domesticated cats share similarities to those of their wild ancestors.

Within 1000 years of the first signs of cat domestication, humans began to transport ancestral cats from place to place, as evidenced by the appearance of fossil evidence of cat and human remains on Cyprus, an island that has no native wildcats, about 9500 years ago [41,43]. It is unclear when *F. s. lybica* became reproductively isolated from *Felis catus*, although archeological evidence suggests that this may have occurred in Egypt approximately 2000 to 4000 years ago [21,44]. While small Egyptian amulets representing cats date from as early as 2300 BCE, the oldest pictorial representation of a cat in a domestic context was believed to be created around 1950 BCE and depicts a cat confronting a rat in a painting in a tomb [8].

It is likely that the phenomenon of group-living in the ancestors of *Felis catus* evolved during the domestication process between 5000 and 10,000 years ago [21]. It is proposed that initially these cats were as solitary and as territorial as their wild counterparts, but because the quantity of rodents and prey available was likely more than necessary to feed a pair of cats and their offspring, the abundance of prey attracted multiple cats [41]. Tolerance to the proximity of other cats may have become an important adaptation, ultimately resulting in decreased aggressive territorial behavior and possibly selection for cooperative tendencies [21].

Evidence suggests that ancestral cats evolved to interact with humans after and due to increased social behavior with other cats. This hypothesis is supported by observations that the repertoire of social signals directed by cats toward people is similar to the species-specific signals used between both adult cats and between mothers and their kittens [21].

While most consider house cats to be fully domesticated, some researchers maintain that cats are only “semi-domesticated” [8,45]. The basis of this argument is that there are limited differences between house cats and wildcats from a morphological, physiological, behavioral, and ecological perspective, as well as the fact that house cats can breed with wildcats and produce viable offspring [46].

Despite the evolutionary success of domesticated cats, two factors may have slowed the domestication process. First, during the second millennium, cats and their owners were intermittently persecuted in Europe, making pet cats a rarity [41]. Second, it has been suggested that the hypercarnivorous diet of cats, which was not always well understood by humans, may have slowed cat domestication since the cat’s basic nutritional requirements were not understood until the 1970s. Until then, cats were often unable to survive and reproduce successfully without supplementing their diet through hunting [41].

### 3.2. Sociality in Domesticated Cats Versus Other Cat Species

The domesticated cat, *Felis catus*, is unique among the other members of the Felidae family in two respects. First, unlike its wild ancestor (*F. silvestris*), the domesticated cat is a variably social animal [1,20,47]. In fact, the social system of free-ranging domesticated cats has been described as “facultative sociality,” with domesticated cats exhibiting flexible social behavior, with the capability of living both alone and in groups [48]. Factors that affect the sociality of domesticated cats include the individual characteristics of the cats (including sex, age, sexual status, body size, temperament, and personality), relationships to conspecifics (kin, familiar individual, or group member), and the environment [48]. The formation of social groups tends to depend on the availability of food, shelter, and mates, although the structure and organization of social groups are not random. Free-ranging domesticated cats that form social groups are more likely to engage in social behaviors with certain members of the group often referred to as “preferred associates.” Although the reasons that certain cats become preferred associates is not entirely clear, studies have shown that preferred associates are typically related and highly familiar to each other [49,50,51].

Some cats display strong bonds with preferential affiliations among group members, while other cat groups show only minimal social interaction [48,51,52]. Cats who do form social groups often exhibit affiliative behavior, including communal denning and alloparental care (among females), while physical fights involving direct contact between cats are infrequent [48]. They are also clearly able to form interspecies relationships with humans [21,53], with domestic cats living as companions in human homes.

Domesticated cats also communicate with members of their species in ways not observed in solitary felids [1,54]. For example, domesticated cats raise their tail as an affiliative behavior [54]. While all felids raise their tail vertically while urine spraying, domesticated cats raise their tail for prolonged periods of time. This can occur during social rubbing, but they also may leave their tail upright even while walking. Adult domesticated cats also use neotenized signals, such as meowing, kneading, and purring, all of which are commonly observed in juvenile felids. However, these signals are routinely used by adult domesticated cats to communicate with humans. These signals are unlikely to be performed by adult undomesticated cat species in captivity, suggesting that undomesticated cats in captivity do not naturally revert to juvenile behaviors as adults. This display of neotenized social signaling is consistent with the observation that neoteny commonly accompanies domestication [55], possibly as a feature of what has been termed “domestication syndrome” [56]. The retention of typically juvenile characteristics into adulthood is also observed in domesticated dogs [57], experimentally domesticated foxes [58] (but see [59]), and possibly humans [60].

## 4. Cognitive Function in Cats: An Overview

### 4.1. The Cat Brain

While significant strides have been made in understanding the cognitive abilities of dogs, cat research lags far behind. However, some inferences can be made regarding cat cognition based upon research in dogs, since the structure of the brain in both animals are consistent with those of carnivorous animals and relatively dissimilar to those of humans [41]. Comparisons between Felidae and Canidae fossils indicate little if any increase in brain size since its split from the Canidae family [16,21]. Relative to the size of their bodies, cats’ brains are less than half the size of those of humans, with much of that difference due to humans’ comparatively large cerebral cortex. While researchers have begun exploring links between neuroanatomical function and cognition in dogs via awake, unrestrained fMRI [61], similar work has yet to be conducted in cats, largely due to challenges in training cats to remain still in the scanner. This motion control training is difficult and time consuming, and the ability of cats to efficiently respond to this training has not been well investigated [62].

Notably, researchers have observed differences in neural scaling rules between primates and non-primate mammalian clades. Primate brains exhibit evolutionarily derived isometric scaling, as both neuron size and density largely remain constant as brain size increases [63]. Human brains are unexceptional in this regard relative to those of other primates and follow these same scaling rules [64]. In contrast, most carnivorans—including both cats and dogs—generally follow commonly observed scaling rules for non-primate mammalian clades, whereby average neuron size tends to increase with brain size [65]. The result of this non-isometric scaling is that neurons become less densely populated in the brain as both brain size and average neuron size increase, such that a carnivoran brain is generally expected to contain fewer neurons than an equivalently sized primate brain [64]. Neuronal composition in carnivorans, including in the domesticated cat, does not appear to be affected by domestication [65].

### 4.2. Perception

Cognitive skills are frequently grouped into various domains that encompass both lower-order and higher-order functions. Examples of common cognitive domains described in animal studies include perception, executive function, memory, physical cognition, social cognition, and attention [66]. While cognitive structure in cats has yet to be fully explored, behavioral assessments continue to be developed to assess various cognitive domains in cats, and these provide the basis for the overview of cognitive function described in the following subsections.

Cat sensation and perception have generally received more research attention than other areas of cat cognition. Studies have examined auditory, olfactory, and visual perceptions and cutaneous sensory mechanisms, as reviewed by Vitale-Shreve and colleagues [3].

Olfaction appears to play a particularly important role from birth and throughout the cat’s life, affecting mother–kitten relationships [67,68] and providing social information about conspecifics [69,70], home ranges [71], and humans [3,72,73]. While the domestic dog’s sense of smell is well known, cats are also macrosmatic, meaning they have a highly developed sense of smell [21,73]. In cats, the vomeronasal organ plays a major role in communication between individuals, with scent marking often used to convey different messages to conspecifics [21,74]. Pheromones are secreted in a variety of contexts, including spatial orientation, allomarking, and sexual communication [73,75]. Although cats have a more diverse range of vomeronasal organ receptors than dogs, both dogs and cats have six major sources of pheromones: the face, pedal complex, perianal complex, genital complex, mammary complex, and urine and feces [73,76]. A deeper understanding of the perceptual world of cats could enable the development of new approaches to the study of feline cognition that could more fully utilize their range of sensory abilities [3].

### 4.3. Physical Cognition and Working Memory

A handful of studies relevant to physical cognition and memory in cats have been conducted, including studies of cats’ understanding of object permanence, ability to navigate around barriers, and working memory for hidden objects [3,77,78,79].

In visual displacement tests, an attractive object or food reward “disappears” behind an obstacle, such as when it is placed inside an opaque container. Cats “pass” the test if they search for the object where it was last seen, suggesting that they understand that the object still exists even when it is not visible [3]. Research indicates that cats easily solve visible displacement tests, demonstrating an understanding of object permanence [3,80,81,82].

In invisible displacement tests, an attractive object is placed in a container and moved behind an obstacle, such as a screen, where the object is then removed from the container when out of the subject’s view [3]. The container, which no longer contains the object, is then shown to the cat. Cats pass the test if they (a) recognize the object is no longer in the container, (b) realize that it was removed behind the obstacle, and (c) search for the object behind the obstacle where it was removed. While most studies indicate that cats cannot solve invisible displacement tests [3,80,81,82], cats are able to pass more ecologically valid versions of the task [81], further supporting the idea that cats understand object permanence.

In transparent-object detour tasks, animals must navigate around a transparent barrier blocking the shortest route to a person or object of interest, such as a food reward. Successful navigation therefore draws not only on physical cognition but also on inhibitory control, as animals must inhibit their desire to move directly toward the reward by temporarily moving farther from the reward to obtain it. In one comparative study of dogs and cats using a V-shaped barrier, dogs were quicker to reach a food reward behind the barrier than cats, and dogs showed an improvement in latency to solve the detour problem across trials, whereas cats did not [79]. Interestingly, cats were more likely to switch sides to solve the detour problem across trials, while dogs were more likely to continue using the same side across trials. However, in this and other problem-solving tasks, it is important to consider that differences in either motivation or cognition can account for differences between species in performance.

Evidence suggests that cats have a working memory for hidden objects that lasts up to at least 1 min and have a highly developed long-term memory [3,78,82,83]. More data are needed to characterize the impact of a cat’s age, breed, and environment on their short- and long-term memory. Additionally, more research into cats’ ability to understand cause and effect and to discriminate between quantities and time is needed [3,84,85,86].

### 4.4. Socialization and Early Cat–Human Interactions

While many domesticated cats live in social groups, either with other cats or with members of other species, research exploring the sociality of cats and how they communicate with others is still in its early stages [3,53]. Studies have shown that free-living domestic cats and colony cats do not form social groups randomly, with preferred associations and closer proximity typically occurring between related individuals [3,50]. Companion cats constantly engage in social encounters with humans and other household pets with varying frequency and degrees of complexity, sometimes forming strong relationships with these individuals [3].

Socialization in cats typically first occurs within the first 2 to 7 weeks of life, a highly sensitive period in cat development [87,88,89,90]. Similarly to dogs, data suggest that cats exposed to frequent handling by multiple humans earlier in life are friendlier and less fearful of humans than cats without this experience [91]. Moreover, kittens socialized to humans earlier in life have also been shown to provide significantly more emotional support to their owners and exhibit less fear toward humans than kittens that were socialized later in their development [87,90].

Earlier handling by humans may also have a physiological influence on kittens. One study demonstrated that kittens who received more frequent handling opened their eyes earlier, left their nest box earlier, and even showed differences in their coat coloration patterns [92].

### 4.5. Cooperative–Communicative Cue Following

Although research into cat social cognition is relatively sparse, cats’ understanding of cooperative–communicative cues has been explored. Cooperative–communicative cue following involves appropriately interpreting the cooperative signals of an agent to obtain some goal (e.g., finding food) [93]. Cats have demonstrated a sensitivity to human cooperative–communicative cues, such as responding to human pointing gestures to locate hidden food [94]. The ability to follow human pointing gestures is also present in domesticated dogs from a very young age [95,96] but interestingly does not appear to be as robust in either wolves [97,98] or non-human primates [99,100]. This pattern of results has led some researchers to hypothesize that domestication may biologically prepare animals for sensitivity to cooperative communication with humans [95,97], but analogous comparisons between domesticated and non-domesticated felids have yet to be performed.

Cats have also demonstrated an ability to use even more subtle social cues from humans to inform behavior, such as the ability to use gazing direction alone, in a cooperative–communicative context [101]. Cats’ success rates using human gaze to locate hidden food appear similarly high to those observed in both primates and dogs [101,102,103]. In contrast to studies involving other species, the use of ostensive vocal utterances by experimenters does not appear to increase cats’ ability to follow the gazing cues of humans to locate hidden food. The investigators suggested that the lack of improvement in cats’ abilities to follow gazing cues accompanied with ostensive vocal cues may have been due to a ceiling effect resulting from high rates of success in following gazing cues prior to introducing ostensive or non-ostensive vocal utterances [101]. Alternatively, they also hypothesized that cats may not be as sensitive to ostension as dogs due to their relatively shorter period of domestication with humans [5].

### 4.6. Trainability

While some researchers argue that dogs are easier to train than cats, some data suggest that this may not be an accurate conclusion. Several studies show that cats are responsive to training via positive reinforcement, learning to perform a range of cued behaviors [104,105]. In contrast to dogs, a majority of cats have been found to prefer human socialization over food [4,75,106]. This suggests that the misconception that cats cannot be trained may stem from a lack of understanding of what motivational stimuli is preferred by individual cats [4]. Additionally, a pilot study found that after conditioning with a secondary reinforcer (beeping sound), cats may have limited success when trained with a secondary reinforcer alone (beep only), and the use of a primary reinforcer alone (food only) was more effective than both the secondary reinforcer and the primary reinforcer paired with a bridging stimulus (beep followed by food) [107]. These results contrast to those observed in dogs, in which several studies demonstrated that training with food as the primary reinforcer paired with a clicker as a bridging stimulus was equally as successful as training with food alone [108,109,110]. Therefore, the feline training process may need to be approached differently than that for their canine counterparts, as adequate motivation may not be ensured by the presence of food rewards alone, and the use of bridging stimuli may not be effective (as is often the case for dogs). Although cats can be trained, more research is needed on how to optimize the cat training process, the results of which must be communicated to cat owners to increase rates of success.

### 4.7. Vocal Communication

When it comes to human interaction, cats have been shown to prefer playing with humans over listening to human vocalization, with preference evaluated via the proportion of time spent making contact with any part of the human (including sniffing, playing, and touching) [4]. Nevertheless, evidence suggests that cats can distinguish between individual humans based on their voices [111] and can discriminate their names from other words [112], even when they are uttered by unfamiliar humans. Interestingly, cats appear to discriminate higher-pitched “cat-directed speech” from normal speech when spoken by their owners but not unfamiliar humans [113]. This pattern appears similar to dogs’ preference for higher-pitched “dog-directed speech” [114] and human infants’ preference for higher-pitched “infant-directed speech” [115].

Conversely, cat vocalizations appear to play an important role in communication with other cats and humans, with research suggesting that cats can make minor changes to their purr vocalizations to change the meaning of the vocalization [3,10]. Cats have also been shown to use vocal cues to direct attention toward themselves but only when humans are looking at them [116]. One study found that cats solicited food from their owners using a “high-frequency voiced component” with frequencies similar to that of an infant’s cry [10].

Vocalizations in domesticated cats living with humans may be different from those of both feral cats and other *F. silvestris* species. For example, researchers have found that the vocalizations of domesticated cats are perceived as more pleasant by humans than those of their ancestor, the African wildcat *F. s. lybica* [116,117]. Evidence suggests that vocalizations of feral cats are typically more aggressive, more frequent, and longer in duration than house cat vocalizations in response to both threatening and non-threatening stimuli [118], suggesting that the degree of human socialization a cat has received can impact their social behaviors and vocalizations [48]. However, the difference in observed aggression between feral cats and house cats may be confounded by spay/neuter status. One study comparing aggression in intact and neutered cats found that the intact cats showed higher frequencies of agonistic behavior [119]. Further research is needed to better understand the meaning and development of cat vocalizations.

### 4.8. Social Referencing and Sensitivity to Human Emotion

The ability of an individual animal to use the emotional reactions of others to evaluate unfamiliar or difficult situations and adjust their behavior accordingly is known as social referencing [120]. Current data suggest that cats are moderately sensitive to human emotion and moderately skilled in social referencing related to humans. While the ability of domesticated dogs to respond to expressions of human emotion is well documented, whether cats possess similar abilities is less well known [5].

When faced with an unsolvable task, cats may gaze at humans for assistance. However, while some cats adjust their behavior based on the response of humans, dogs are more likely to adjust their behavior based on the response of humans than cats [1,94]. Additionally, cats appear to glance at their owner less frequently than dogs when faced with a detour task [79]. Still, some data do suggest that similar percentages of cats and dogs look referentially toward their owners when faced with an unfamiliar object [1,121,122]. While these results appear to be conflicting, it has been proposed that cats may have explicit uses for gazing behavior that differ from dogs. For example, they may use gazing when they are in a state of uncertainty or fear (as when faced with an unfamiliar object) but may be less likely to display this behavior when problem solving (as when faced with an unsolvable or detour task) [3,123].

One study reported that cats do not appear to display the same level of response to extreme human emotions (happiness and anger) demonstrated through facial, postural, or vocal cues as dogs but did show significantly more positive behaviors toward their owners in the happy emotion condition versus the angry emotion condition, such as having a relaxed posture, ears facing forward, and more time spent in contact with their owner [5]. Another study found that cats were able to recognize and respond to human emotional signals, as represented by facial expression and vocalizations [124].

Cat behavior can be influenced by human moods, with the previously mentioned study indicating that cats exhibited a higher stress/anxiety score (as determined via the number of behaviors displayed indicating stress and/or anxiety) when exposed to human anger vocalizations [124]. Additionally, cats are more likely to engage in allorubbing with owners in depressive moods and approach owners who reported feeling extroverted or agitated [125,126]. These studies demonstrate that cats may have a greater understanding of and sensitivity to human emotions than previously expected.

### 4.9. Attachment to Humans

Cats, like dogs, often form an attachment to their owners, which can be described as the relationship, or bond, between individuals [127,128]. A secure attachment is characterized by spending more time in physical contact with their owners than with strangers while also spending more time moving and exploring in the presence of their owners [129]. Insecure attachments can be characterized as insecure-ambivalent, insecure-avoidant, or insecure-disorganized [128]. When their caregivers return after a brief separation, cats with secure attachments exhibit low levels of distress while those with insecure attachments remain stressed and display attention-seeking or avoidance behaviors [128]. The development of secure attachments to humans could facilitate the development of social cognitive skills that might increase cats’ ability to successfully navigate human environments [128,130]. In a study of 70 kittens aged 3 to 8 months, 64.3% were classified as securely attached to their caregivers and 35.7% were insecurely attached [128], similar to the frequencies observed in children and dogs [128,131,132]. The style of an individual cat’s attachment to their human caregiver appears to be relatively stable through adulthood [128].

Some pet cats may show signs of separation anxiety in the absence of their caregivers. Distress upon separation can be seen in cats with both secure and insecure attachments to their caregiver and is characterized by inappropriate urination and/or defecation, increased vocalization frequency, destructiveness, aggression, excessive grooming, anxiety, and depression [128,129,133]. A survey of 130 owners of 223 cats found that 13.45% of cats exhibited at least one of the behavioral characteristics indicative of separation-related problems, suggesting that separation anxiety may be an issue for a large population of pet cats [134].

How cats interact with people appears to be influenced by the attentional state of the person, with both pet and shelter cats spending more time in proximity to people who are attentive [128,135]. As discussed above, many cats even prefer social interaction with humans over other rewards, including food or toys, although more work is needed to assess cats’ preferences in similar and alternative contexts [4,48].

### 4.10. Personality

Personality is described as an individual’s collection of consistent behaviors in given contexts [136,137]. A pet’s personality is closely linked to their welfare and has been shown to impact a variety of health outcomes [22]. Cat owners’ lack of knowledge or misinterpretation of cat personality expression and natural behaviors has been cited as a factor that contributes to behavioral problems, including inappropriate elimination, aggression, and destructive activity, which weaken the owner–cat relationship [138,139,140,141,142,143]. Therefore, a greater understanding of cat personality and appropriate cat behavior may reduce the rates of pet cat relinquishment [139].

Cat owners and researchers investigating cats consistently indicate that cats have clear, identifiable, and stable personalities, although personalities among individuals can vary widely [3,144,145,146,147]. Common personality traits exhibited by cats that are described in the literature include boldness, shyness, easy-goingness, trustingness, timidity, and nervousness.

Bradshaw and colleagues divided cat personalities into three types, including individuals who are either (1) sociable, confident, easy-going, trusting, and bold who initiate friendly interactions; (2) timid, nervous, shy, and unfriendly; or (3) aggressive [22,53]. Another study reported six main personality dimensions as perceived by owners in a sample of 416 adult cats, including playfulness, nervousness, amiability, dominance, demandingness, and gullibility [139].

Evidence suggests the personality of cats is influenced by genetic, social, and environmental factors [21,139,148,149,150,151,152]. The observation that differences in cat personalities arise in kittens as young as 5 to 6 days old, with kittens aged 3 to 4 weeks already developing relatively stable behavioral differences, suggests a possible genetic component to the development of cat personality [151], similarly to what has been described in dogs [153]. This hypothesis is supported by the finding that male cats described as “friendly” typically sired “friendly” offspring even though they had never encountered each other [3,90,146,154]. The relationship between social and environmental factors and cat personality is reinforced by the observation that the social structure of cats living in groups relies on characteristics of individuals in the group, with the more aggressive individuals tending to be higher in the social hierarchy than those exhibiting submissive or defensive behavior [21]. Similarly, the presence of other cats in a household has been shown to have an impact on several personality traits in pet cats [139,149,155], as evidenced by a survey-based study that showed that cats living in multi-cat households had higher owner-reported sociality and aggression toward humans [155]. More research is needed regarding the extent to which social groups that exist in both free-roaming and household cats influence personality, yet these preliminary studies reveal that a clear link exists between social interaction and personality in cats. The environment a cat lives in and is exposed to has an impact on their personality, with the long-term presence of stressors and/or stimuli significantly influencing the behavioral patterns expressed [150,152].

Studies investigating the effect of neutering status on feline personality have reported mixed results. While intact cats have been shown to be more aggressive compared to neutered cats [119,156], this has not been found in all studies [149,157]. More work is needed to determine the relationship between neutering status and personality, if one exists.

### 4.11. Emotion and Mental Experiences

Relatively little is known about emotion in cats. Some researchers believe that cats may not be capable of experiencing complex emotions that require a sense of self or an understanding of the past, present, and future, such as guilt, pride, or grief [41]. The ability of non-human animals to engage in this sort of “mental time travel” is relatively contentious given the difficulty of operationalizing behavioral criteria relevant to studying it. Similarly difficult to elucidate in non-human animals is the potential capacity for episodic memory or the ability to mentally recreate past experiences [158]. Researchers have turned to operationalizing behavioral criteria relevant to “episodic-like memory” to navigate these challenges in varying species, including rats and scrub jays [159,160], although the topic remains largely unexplored in cats.

Some cats may appear to grieve for missing companions, but this behavior may be explained by lower-level mechanisms, such as their reactions to the lingering odor of the absent companion. Simpler emotions, such as anger, affection, fear, and anxiety, are argued to exist more broadly across mammals, including cats, and these emotions likely drive much of a cat’s behavior. Nevertheless, more work is needed to elucidate the emotional capacity of cats and understand how these emotions are expressed [41].

### 4.12. Stress

Stress can be described as the physiological and behavioral response to a stimulus, threat, or perceived threat to homeostasis [161]. The ability to produce adaptive responses to stressors is a natural survival mechanism. Cognitive function can be impacted by stress, which may present as chronic or acute and may be caused either by aversive emotional states such as fear or anxiety or by physiological status, such as disease and inflammation [162]. As reviewed by Calvo and Gutierrez-Garcia, acute stress may provide a favorable or detrimental impact on cognitive processes connected to memory, learning, and information processing [163]. Chronic stress generally has a deleterious effect on cognition and may result in damage to brain structures and an increased risk of dementia in humans [164]. Signs of stress in domestic cats include behavioral changes, such as changes in normal activity levels, changes in appetite, changes in vocalization patterns, avoidance, elimination problems, aggression, and abnormal repetitive behaviors, such as feline hyperesthesia syndrome, overgrooming and psychogenic alopecia, and pica [165]. Other signs of stress in cats include gastrointestinal problems, including diarrhea or vomiting, the development of feline interstitial cystitis, atopic dermatitis or acral lick dermatitis, and anorexia. Questionnaire-based tools for evaluating stress in cats include the Cat Emotional Scale [166], Cat Stress Score [167], and portions of the Feline Behavioral Assessment and Research Questionnaire (Fe-BARQ) [168].

As social generalists, domesticated cats show a wide range of sociality and preferences for social behavior, which may in turn influence when cats become stressed. For example, some pet cats become stressed when living with other cats, while others become stressed when living without other cats [48]. Evidence suggests that cats show a lower frequency of stress-related behavior when their owners are present compared to when their owners were not present, including a reduction in the frequency of distress vocalizations when their owners returned after time away [169]. In a study of 43 cats, 83% engaged in directed allorubbing when they were reunited with their owners, supporting the idea that this behavior may be calming and associated with affiliation as well as a strategy for cats to mark their owner as a part of their “group” [169,170].

### 4.13. Aging

Cognitive function is an important contributor to the welfare of cats, and factors such as aging and the feline immunodeficiency virus can impair feline cognitive abilities [171,172,173,174]. It is increasingly evident that cats experience age-related neuropathologic changes that parallel those observed in humans with Alzheimer’s disease, though some key differences do exist, such as the reported absence of neurofibrillary tangles in cats [175]. Because cats are living longer today than ever before due to advances in feline nutrition, veterinary medicine, and owner awareness of feline health, the prevalence of age-related feline cognitive decline is increasing. In fact, one study reported that 28% of cats aged 11 to 14 years without systemic illness experienced at least one behavioral sign attributable to cognitive dysfunction syndrome (CDS) and this increased to 50% among cats aged 15 years of age and older [176]. Similar increased prevalence of CDS with age is also observed in dogs, with many overlapping behavioral changes implicated [177]. Despite the prevalence of CDS in the feline population, associated behavioral changes are often subtle, and when identified, they may be dismissed as a normal result of aging, leading to a lack of appropriate veterinary care [178].

Feline CDS is associated with age-related brain deterioration and involves specific behavioral changes that cannot be attributed to any other medical condition [179]. Pathological changes associated with CDS include neuronal loss, cerebral atrophy, widening of sulci, microhemorrhages or periventricular infarcts, an increase in reactive oxygen species leading to oxidative damage, deposition of beta amyloid in extracellular plaques, and a decline in cholinergic function [175]. Collectively, these changes may contribute to declines in working memory, alterations in motor function and REM sleep, and other signs of CDS [175].

CDS is both difficult to diagnose and difficult to treat in cats. The syndrome of CDS has been compared with Alzheimer’s disease (AD) in humans, and as with AD, the pathophysiology of CDS is largely unknown, although compromised cerebral blood flow and damage from chronic free radicals are believed to play roles in both conditions [179,180,181]. The behavioral changes of CDS in cats are summarized in the acronym VISHDAAL and include increased vocalization, altered social interactions, changes in sleep/wake patterns, house soiling, disorientation, changes in activity, anxiety, and learning and memory deficits (Table 1) [178]. As these behaviors occur mostly in the home environment, it can be difficult for veterinarians to directly assess them, often leading to reliance on pet parent questionnaires, interviews, or reports when diagnosing CDS.

Objective data on the impact of age on cognitive function in cats are limited [182]. Preliminary data demonstrated differences in age-related performance on both discrimination and reversal learning portions of a T-maze task [183], with senior cats showing impairment versus younger, healthy adults [182,184]. However, this trend was not observed in all cats or across all tasks, with some older cats demonstrating improved performance on some cognitive tasks. The varying performance on cognitive tasks within the aging cat population may be related to the extent of cognitive decline in the animal and whether or not the animal has signs consistent with CDS or “normal” aging. More work is needed to investigate and distinguish what is considered a “normal” change in cognitive function in cats during the natural aging process and what is indicative of CDS. It is also crucial to consider the effect of illness and/or pain on the manifestation of behavioral changes, as these medical issues may be the root cause of the behavioral changes observed [173,185].

## 5. Nutrition, Behavior, and Cognition

Despite the fundamental role of nutrition in the development of the brain and the impact of poor nutrition on cognitive function and behavior in animals, research into the association between nutrition and cognition in domestic cats is only in its early stages.

### 5.1. Food Preferences

Wild and free-roaming cats are solitary hunters and typically feed on small prey. Given the size of their prey, cats often require multiple kills per day to meet their energy requirements [186]. The prey is usually eaten immediately, which may explain their preference for food to be at body temperature. When eating larger prey, they typically prefer the flesh (muscle tissue), fat, and internal organs other than those in the digestive tract.

Food preferences of cats are also strongly influenced by the food preferences exhibited by their mothers and the foods they were exposed to during pregnancy and lactation [33,186,187,188,189]. Like dogs and other mammals, cats also rely on smell and taste to detect and select foods. While both senses help them detect the freshness and safety of food, taste is the more dominant sense in influencing their preferences [26,186,187,190]. Although their sense of taste is similar to that in other mammals, who can detect salty, bitter, and sour stimuli and amino acids, they lack taste receptors for sweet carbohydrates and thus show no preference for carbohydrate sweeteners, like sucrose [191,192].

Studies have shown that many cats prefer foods with a strong “umami” or savory flavor, which can be associated with foods with high concentrations of amino acids [186,193,194]. However, they do show a preference for some amino acids over others, appearing to reject amino acids that taste “bitter” to humans, such as L-arginine, L-tryptophan, L-isoleucine, and L-phenylalanine, and prefer amino acids that taste “sweet”, such as L-proline, L-cysteine, and L-lysine, among others [26,186,189,191]. They also tend to reject foods with monophosphate nucleotides, which can accumulate in mammalian tissue after death, which may explain their tendency to refuse decaying flesh [26,186,189].

When given multiple food options, studies of cat preferences have reported that they base their food choices on sensory considerations (how the food smells, tastes, and feels in the mouth) as well as past experience [195]. Some cats show a strong individual preference for novel foods (neophilia), although this effect is typically short-lived when observed. In contrast, many cats tend to be more neophobic in some instances and may reject unfamiliar foods [196], especially when ill. When a specific food is associated with a negative experience, whether physical, emotional, or psychological, cats may develop an aversion to it, and they will continue to avoid the food in the future [26,195,197]. Compared to dogs, cats are more selective eaters and can detect small differences in the composition of the food they consume [26,186,187]. If a food change is necessary, it is important for cats (especially those that are sick) to be slowly and gradually transitioned onto the new food to avoid rejection [198].

Interestingly, research suggests that cats are able to consume a “target intake” of protein, fat, and carbohydrates when given a choice of foods differing in macronutrient composition [186,199,200]. For example, when differing diets with varying protein and carbohydrate contents were fed to cats, they were able to regulate their macronutrient intake to obtain approximately 53% of energy from protein and 11% from carbohydrates [194]. However, this dietary selection may largely be due to the high palatability of meat-based food for cats. Prior to controlling for palatability, cats exhibited a preference for high-protein food. After adjusting for palatability through the use of flavor enhancers or ingredient substitutions, cats chose to consume foods with a higher level of carbohydrates, with 43% of their calories coming from carbohydrates and 30% of calories coming from protein [199], although younger, leaner cats tended to consume more protein than older cats. Therefore, when palatability is not a factor and cats are allowed to choose their macronutrient intake based on physiological needs, cats may choose to obtain a majority of their calories from carbohydrates.

### 5.2. Impact of Diet on Behavior

Given that behavior is regulated by neurotransmitters and hormones, dietary factors that affect the availability of their precursors can influence their production and the behaviors that they influence [201]. For example, in both humans and other animals, deficiencies of vitamins and minerals have been associated with mood disorders, including anxiety and aggression [202,203,204].

Tryptophan and tyrosine are the precursors to serotonin and dopamine, two neurotransmitters that play an important role in learning, impulse control, and emotion [201,205]. Lower levels of serotonin have been shown to be associated with aggression in many species, and a diet high in tryptophan, in some studies, has been shown to aid in decreasing aggression and improving an individual’s ability to cope with stress [201,205,206]. For example, when fed a high-protein diet without L-tryptophan supplementation, dogs had higher dominance aggression scores compared to when they were fed a high-protein diet supplemented with L-tryptophan or a low-protein diet with or without L-tryptophan supplementation [207]. The effects of tryptophan supplementation on feline aggression are unclear.

Several nutraceuticals have exhibited preliminary but promising results for the treatment of signs of fear, stress, or anxiety in cats, including alpha-casozepine and L-theanine [166,201,208,209] either alone or in combination with other ingredients, including the herbs *Magnolia officinalis* (also known as magnolia bark extract) and *Phellodendron amurense*, and concentrated whey protein (Solliquin) [201,210]. Diets supplemented with milk protein hydrolysate (including α-casozepine) and L-tryptophan may also reduce feline anxiety [178,201,211,212].

**Figure 1:**
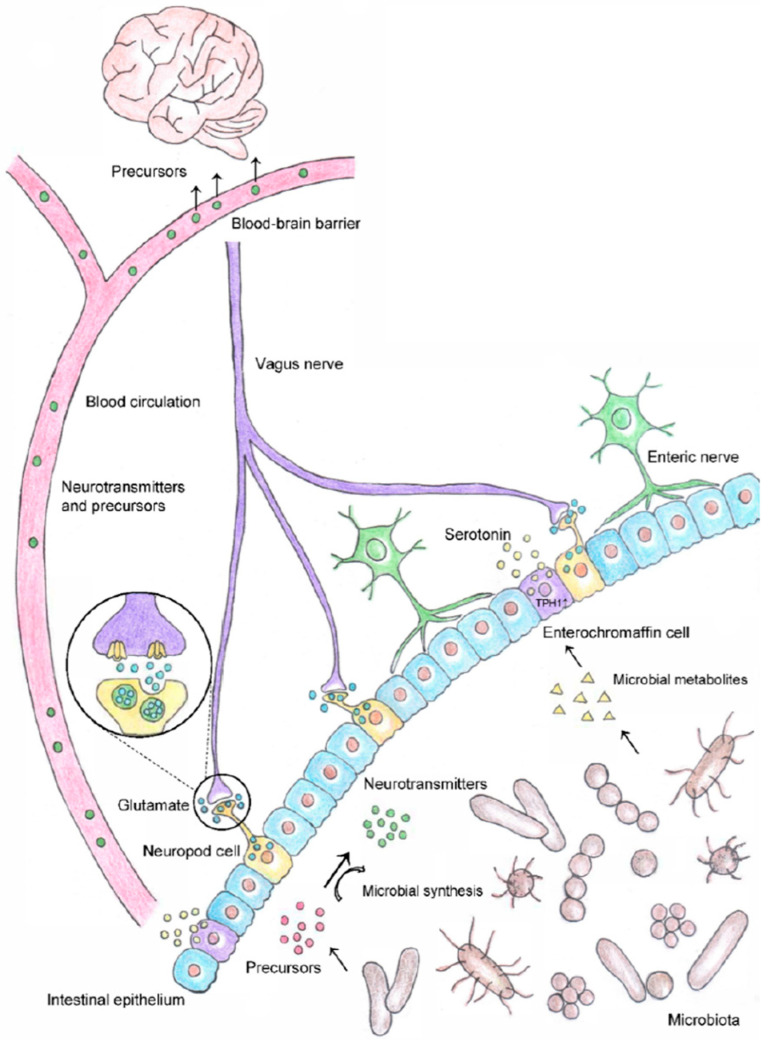
Gut microbial-mediated neurotransmitter synthesis in humans and its potential impact on cognition [213]. Reproduced from Chen Y et al. *Nutrients*. 2021;13(6):2099. doi: 10.3390/nu13062099, available under a Creative Commons Attribution 4.0 License.

Increasing evidence suggests that the gut microbiome can also influence cognition and behavior (Figure 1) [201,205,213,214]. The brain–gut–microbiota axis consists of the central nervous system, the neuroendocrine system and the neuroimmune system, and the autonomic and enteric nervous systems, as well as the intestinal microbiota [201,215]. The nerve fibers that integrate these systems enable bidirectional communication between the brain and the GI tract [201]. The hypothalamic–pituitary–adrenal (HPA) axis is activated when cats are in physical or psychological stress, and a dysregulated or overactive HPA axis can produce excessive stress hormones, increased inflammation, and altered gut permeability, motility, and secretion [201,215]. These changes may result in diarrhea or other signs of GI distress, and even dysbiosis, or an imbalance in the gut microbiome. Stress that leads to increased gut permeability can result in pathogenic bacteria crossing the epithelial barrier, resulting in an inflammatory immune response that further activates the HPA axis [201,215].

Accumulating data also suggest that while the HPA axis can impact the composition of the gut microbiome, the opposite may also be true: a healthy gut microbiome is important for the normal development and function of the HPA axis [201,215]. The gut microbiome has been reported to influence CNS function by activating stress circuits, which contributes to anxiety and depression [201,216]. Some microbes may also produce neurotransmitters and/or their precursors, which can interact with and impact the HPA axis and responses to stress [217]. Many problematic behaviors in dogs and cats result from fear and anxiety, and given the bidirectional relationship between the gut microbiome and the HPA axis, the gut microbiome may play a role in the onset of these behaviors.

### 5.3. Dietary Management of CDS: An Overview

According to the American Association of Feline Practitioners (AAFP), recommended strategies for managing CDS in cats include client education; environmental optimization; supplements with essential fatty acids, antioxidants, and B-vitamins; and pheromones [218]. If these interventions do not improve signs of cognitive dysfunction, selegiline, a monoamine oxidase inhibitor that increases the level of dopamine present in the brain, may be beneficial, though selegiline should not be given without a prescription and supervision by a veterinarian. Individualized combination therapy is most effective and may improve the brain function, longevity, and quality of life of cats with CDS [218,219].

Although research into the cognitive and behavioral impact of different nutrients, including fatty acids, amino acids, and medium-chain triglycerides (MCTs), in cats is extremely limited, considering results from studies in other species may be informative [178,220]. In humans, brain aging, stroke, and dementia have been linked to several risk factors, including DHA deficiency; low levels of vitamin B6, B12, and folic acid; high homocysteine; high blood pressure; cerebrovascular lesions; increased oxidative stress; and chronic inflammation [220]. While research still needs to confirm these risk factors in cats, it is possible that nutrients or supplements that target these risk factors may reduce the risk of CDS or slow its progression in adult cats.

Developing diets to reduce the signs of CDS in cats has been challenging [178]. Several studies in dogs with CDS have shown behavioral improvements and reduced deposition of amyloid in the brain with the use of supplements or diets with a range of combinations of vitamins, antioxidants, essential fatty acids, and other potentially useful components [179,220,221,222,223,224,225,226,227,228,229,230]. To our knowledge, no nutritional studies have been conducted in cats diagnosed with CDS; therefore, very little is known about the nutrition that may be beneficial for cats with CDS.

Dietary management of CDS, if used appropriately, is likely to be safe and have relatively few side effects compared with pharmacological agents. Many pet supplements and diets with nutritional supplementation may be palatable for cats and easy to ingest, which would improve adherence and the likelihood of favorable outcomes [201].

### 5.4. Role of Antioxidants

Oxidative stress results from an imbalance between prooxidants and antioxidants. Prooxidants, including reactive oxygen species (ROS), damage cells and tissues through oxidation, while antioxidants are enzymes (e.g., superoxide dismutase or catalase), nutrients (e.g., vitamin C, vitamin E, or beta carotene), or other compounds that act by either binding to, preventing the formation of, or capturing free radicals on reactive oxygen species [231,232]. Although the body produces numerous compounds that function as antioxidants, endogenous antioxidant capacity decreases with age, likely increasing oxidative stress in the body [232]. Given its high metabolic rate, large oxygen demand, and relatively low concentration of endogenous antioxidants, the brain is particularly susceptible to oxidative stress [233].

Increased production of free radicals with aging can lead to neuronal dysfunction and, ultimately, neuronal death [234]. In the brains of older dogs, carbonyl groups accumulate, which is an indication of oxidative damage to proteins and a reduction in endogenous antioxidant activity [234,235,236,237,238,239]. Increased oxidative damage can also be measured by increased lipid peroxidation and may result in damage to brain DNA or RNA [234,240,241]. Aged dogs have also shown mitochondrial dysfunction and oxidative damage that is frequently observed in humans with age-related neurologic dysfunction [234,236].

Oxidative damage is likely associated with declines in cognitive ability in some domains [234]. Studies of aged beagles have shown that higher protein oxidative damage and lower endogenous antioxidant activity are associated with impaired discrimination learning, reversal learning, and spatial memory [234,238]. It is expected that oxidative damage to the brain also contributes to declines in brain function and cognition in cats, although more research in this area is needed.

Consequently, the inclusion of antioxidants and eicosapentaenoic acid (EPA) in dietary supplements may also help reduce oxidative stress-induced damage and low-grade inflammation, both of which can contribute to brain aging and dementia in humans [220,242,243]. In fact, dietary supplementation with antioxidants has been shown to increase various aspects of cognitive function and slow cognitive decline in multiple mammalian species [232,244,245,246,247]. Combinations of nutrients may result in more promising effects than the use of single nutrients [232,248].

Because antioxidants serve to scavenge or inhibit the formation of ROS in the body, it is expected that antioxidant supplementation in the diet can help protect the brain from oxidative damage and, in turn, reduce the risk and progression of cognitive decline in cats. While numerous studies have evaluated the effects of antioxidant supplementation in the diet on the brain health and behavior in aging dogs, more research on the cognitive impact of antioxidants in cats is needed [230,231,232,244,247,249,250,251].

### 5.5. Role of Omega-3 Fatty Acids

Omega-3 polyunsaturated fatty acids (PUFAs), including EPA and docosahexaenoic acid (DHA), are critical to brain functioning [201,252]. DHA, the predominant lipid found in the brain, plays a pivotal role in the production of phosphatidylserine (PS), a compound found in the cell membrane of neurons that helps activate signaling pathways in the neuronal system [201,253]. Omega-3 fatty acids also play a role in mediating inflammation [253], which has been suggested to be a major contributor to declining brain function in humans and cats [180]. The inclusion of omega-3 fatty acids in the diet may therefore support brain health by protecting the integrity of cellular lipid membranes in the brain and contributing to a reduction in brain inflammation [180,201].

Supplementation of DHA and EPA through the addition of fish oil has been reported to improve cognitive function in people and middle-aged rodents and slow cognitive decline in humans exhibiting mild cognitive impairment in some studies [220,254], although the optimal levels of DHA and EPA for maximal brain benefit have yet to be determined.

As with any drug or supplement, cats that receive omega-3 fatty acids in very large amounts could experience adverse effects, including altered platelet function, GI effects, impaired wound healing, and lipid peroxidation [201,255]. The data are insufficient to establish a safe upper limit for cats, but 2800 mg/100 kcal has been suggested as the upper limit for the EPA and DHA in combination in dogs [201,255].

### 5.6. Specific Diets Evaluated in Aging Cats

While no specific diet has been designed for cats with CDS, some diets involving certain ingredients or interventions have shown promise in improving the cognitive functions affected by CDS. For example, a diet supplemented with fish oil, antioxidants, arginine, and B vitamins showed a beneficial effect on various measures of spatial and size discrimination learning and reversal learning in middle-aged and older cats, suggesting that such a diet may be able to mitigate the deleterious effects of CDS [220]. However, this study did not specifically evaluate cats diagnosed with CDS, so it is unclear if similar effects would be observed in this population.

Longitudinal studies have shown that a diet containing antioxidants, omega-3 and omega-6 fatty acids, and dried chicory root was associated with increased longevity in cats [178,256,257]. Similarly, a randomized controlled study that compared the impact of a therapeutic food containing added antioxidants, including vitamin E, ascorbic acid, beta carotene, and sources of n6 and n3 fatty acids from chicken fat and fish oil, carnitine, and phytonutrients, with any control non-therapeutic food in 105 cats aged ≥9 years showed improvements in health and behavior measures with the therapeutic intervention [258]. The pet owners of cats who were randomized to the therapeutic food reported improved vitality, coat texture, interactions with family members, and ability to run and play in their cats compared with cats assigned to the control group and versus baseline.

An “anti-aging” formula containing a protein source (derived from chicken, egg, or corn gluten meal), a carbohydrate source (millet, brewers rice, and/or oat groats), a vegetable source (carrots, spinach, and/or tomato pomace), and a fruit source (citrus pulp) has also been developed and patented for dogs and cats [259]. In adult dogs, this food has been shown to increase levels of ceruloplasmin, peroxiredoxin-1, and proteasome, all of which are involved in the defense against oxidative stress and are reduced in elderly animals. The therapeutic food also reduced the rate of muscle and cartilage degradation and improved immune responses, mineral transport, and gastrointestinal health.

Although no diet has been developed specifically for cats with CDS, a range of products are now available that contain antioxidants, fish oil, and other types of nutritional additives or interventions [182]. A preliminary study of 46 cats found that cats fed a diet supplemented with tocopherols, L-carnitine, vitamin C, beta-carotene, docosahexaenoic acid, cysteine, and methionine exhibited increased activity levels after 30 days compared to cats fed the control food [260].

Finally, a study evaluating a test food developed with ingredients chosen for their purported anti-aging benefits, including a fiber blend, natural antioxidant polyphenols, fish oil, vitamins, and minerals, reported that cats receiving the test food showed lower levels of metabolites associated with detrimental processes (e.g., uremic toxins) and aging and higher levels of metabolites associated with beneficial processes (e.g., tocopherols) compared with the control food [261].

The supplementation of arginine, B vitamins, L-carnitine, and MCTs have been studied for their potential effects on brain health, but the individual impact of each nutrient on the brain as a result of supplementation has not been tested in cats specifically. Among these nutrients, arginine, which acts as a precursor to nitric oxide, enhances nitric oxide synthesis and has been shown to help maintain normal blood pressure, circulation, and cognition in humans [201]. Vitamins B_6_ and B_12_ and folic acid all play important roles in CNS health. Low levels of vitamin B_12_ and folic acid lead to elevated levels of plasma homocysteine, which is associated with an increased risk of cognitive impairment in humans [201]. Vitamin B_6_ also supports normal brain development and function and exhibits neuroprotective effects [201]. L-carnitine is necessary for normal mitochondrial function and helps regulate ketogenesis. L-carnitine can act as an antioxidant and has free radical-scavenging activity and has been shown to be neuroprotective [262]. Lastly, MCTs have the potential to increase the levels of ketones in the brain and have been demonstrated to improve memory in humans with cognitive dysfunction, though studies have shown that MCTs may not be highly palatable in cats in some instances [201,263]. Much work is needed to assess the efficacy of these nutrients on brain health and cognitive function, particularly in cats.

### 5.7. Other Nutraceuticals and Functional Ingredients for CDS

Nutraceuticals have been defined as a nondrug substance that has been purified or extracted and administered orally with the intent of improving the health or well-being of the individual using it [264]. In the United States, nutraceuticals are legally considered food and are therefore not required to demonstrate the efficacy, safety, and tolerability that is needed for traditional pharmaceutical products, given that the product does not claim to treat, prevent, mitigate, or cure disease [265]. Consequently, oversight of nutraceuticals is generally lax. However, in the United States, if the nutraceutical is included as an ingredient in a complete and balanced diet, it must meet ingredient guidelines determined by the FDA and AAFCO [266]. Additionally, in the European Union, nutraceuticals must be in compliance with Commission Regulation No. 68/2013 when they are included as feed ingredients, and if included as an ingredient in a complete and balanced diet, nutraceuticals must meet the nutritional guidelines of FEDIAF, the European Pet Food Federation [267].

Certain functional ingredients that exist as both nutraceutical products or are included in therapeutic diets may benefit feline cognitive function and cats with CDS. S-adenosyl-1-methionine (SAMe) is a nutraceutical that functions by helping to maintain the fluidity of cell membranes and enhance the production of glutathione, an antioxidant [268]. In dogs, SAMe has been reported to increase activity and awareness [179,225,269,270]. In cats, SAMe also may provide some benefit in improving executive function but was ineffective in improving either short- or long-term memory [179,269]. These early studies have reported that the use of SAMe tosylate supplements have improved executive function in older cats but did not show significant improvement in cats in the bottom half of performers at baseline, suggesting that this intervention may be best used during the early stages of cognitive decline (i.e., when cognitive impairment is less advanced).

MCT supplementation has been shown to improve some aspects of cognitive function, including spatial-working memory, problem-solving ability, and owner-reported trainability in dogs with epilepsy [271], as well as spatial learning, reversal learning, and attention in aged beagles [228]. It has been suggested that diets supplemented with MCTs may benefit cats with CDS, but this intervention has been largely unexplored and requires further evaluation [182,272].

Complementary therapies, such as melatonin, plug-in pheromones, L-theanine, milk protein hydrolysate, essential oils, and amino acid/herbal combinations, may help in correcting sleep/wake cycles and reducing anxiety [175,178,182,273].

Unfortunately, once CDS is severe, significant environmental or nutritional changes may have a negative effect [178,274]. Due to the nature of the disease, affected cats tend to respond particularly poorly to changes, whether the changes are related to their diet, environment, or daily routine. At this stage in the disease, change should be kept to a minimum and, when required, should be made slowly and with reassurance.

### 5.8. Drug Therapy for CDS

While many drugs are currently used in cats with CDS in an off-label capacity, no drugs are currently licensed for use in cats with CDS. A veterinarian should always be consulted prior to the administration of any medication.

Psychotropic medications can play a valuable role in many cats with CDS but may be less preferred by some caregivers and may not be ideal for every cat, especially frail geriatric cats [201]. Selegiline and propentofylline have been used in cats with varying degrees of success [179,275,276], while anxiolytics and antidepressants have been used to manage symptoms. However, selegiline should not be given with serotonergic medications. Controlled trials on the use of these medications in adult cats with CDS are needed [179].

In some cats, melatonin may help restore sleep cycles, while telmisartan, an angiotensin receptor blocker, has shown reduced neurodegeneration in rats and beneficial effects in people with hypertension and AD [179,277,278].

Given the reduced cholinergic function and decreased numbers of cholinergic neurons in cats with CDS, drugs that enhance cholinergic transmission, such as the cholinesterase inhibitor donepezil, may improve CDS symptoms, although its use has not been evaluated in cats [178,279]. Much more research is needed regarding the safety and efficacy of these medications in cats.

### 5.9. Opportunities in Feline Cognition and Nutrition

To improve the welfare of aging cats, additional approaches that prevent, slow, manage, or reverse CDS symptoms are needed. Because neurons cannot be replaced in sufficient quantities to restore normal brain function after they are lost, there should be a greater focus on CDS prevention, possibly using nutrients or bioactive agents that combat neurodegeneration [220]. To the authors’ knowledge, no studies comparing the relative efficacy of individual diets, nutrients, or supplements in cats diagnosed with CDS have been conducted, and there is little evidence surrounding the impact of combining multiple options [201]. Nevertheless, combination therapy is expected to most effectively manage cats with CDS [218,219].

Still, more research is needed to determine the most appropriate combination of nutrients and dietary factors and the optimal concentrations of these nutrients to maximize their beneficial effect on cognitive function, brain health, and behavior in cats. Additional research on the role of the microbiome and the brain–gut–microbiome axis on feline cognition and behavior is also needed.

## 6. Conclusions

An animal’s welfare can be “determined by whether, and how closely, it is able to perform behaviors that are ‘natural’” [75,280]. The social, physical, and mental health of animals is closely tied to their welfare and must be evaluated consistently and accurately. Therefore, we must strive to better understand the social behavior, cognition, and physiology of domestic cats to provide an environment and nutritional profile that allows pet cats to have the best possible quality of life.

As we continue to learn more about the history and cognition of the domesticated cat, we may find ways to utilize the cat’s social tendencies and cognitive skills to improve their welfare and help them feel more secure in human spaces. Many research questions related to cat cognitive function and the influence of nutrition on cat cognition are largely unexplored. For example, to what extent do cats change their social behaviors to communicate with humans? How do changes in diet affect cat cognition and behavior? How do lifetime experiences and related changes in diet influence cat cognition, especially as they age? What differences in cognitive function exist between owned cats with varying outdoor exposure, community cats, and truly feral cats?

Answers to these questions may lead to improved human–cat relationships by providing insight into optimal strategies for interacting with, feeding, and caring for cats, which may strengthen the cat–owner bond and, ultimately, reduce feline relinquishment.

## Figures and Tables

**Table 1:** Clinical signs of CDS represented by the mnemonic VISHDAAL [178].

Letter	Sign
V	Increased **V**ocalization, especially at night
I	Altered social **I**nteraction with the family and/or pets
S	Changes in **S**leep/wake patterns
H	**H**ouse soiling
D	Spatial and temporal **D**isorientation
A	Changes in **A**ctivity (e.g., aimless wandering)
A	**A**nxiety
L	**L**earning and memory deficits

## Data Availability

No new data were created or analyzed in this study. Data sharing is not applicable to this article.
